# A Case of Retroperitoneal Castleman's Disease and an Update on the Latest Evidence

**DOI:** 10.1155/2014/643746

**Published:** 2014-11-05

**Authors:** Eleftherios Spartalis, Petros Charalampoudis, Apostolos Kandilis, Antonios Athanasiou, Petros Tsaparas, Athanasios Voutsarakis, Ioannis D. Kostakis, Dimitrios Dimitroulis, Evanthia Svolou, Penelope Korkolopoulou, Nikolaos Nikiteas, Gregory Kouraklis

**Affiliations:** 2nd Propedeutic Department of Surgery, University of Athens Medical School, General Hospital “Laikon”, Agiou Thoma 15b, Vasilissis Sofias 49, Kolonaki, 106 76 Athens, Greece

## Abstract

Castleman's disease is a benign lymphoproliferative condition with three distinct histological subtypes. Clinically it presents in either a unicentric or multicentric manner and can affect various anatomic regions, the mediastinum being the most frequent location. We herein present a rare case of unifocal retroperitoneal mass proved to be hyaline vascular Castleman's disease. We perform a review of the current literature pertaining to such lesions, focusing on the management of the various clinical and histological variants of the disease. Surgical excision is the treatment of choice for unifocal Castleman's disease.

## 1. Introduction

Castleman's disease is a benign lymphoproliferative condition with three distinct histological subtypes: hyaline vascular, plasma cell and mixed. Clinically it presents in either a unifocal or multifocal (multicentric) manner and can affect various anatomic regions, the mediastinum being the most frequent location. We herein present a rare case of unifocal pararenal mass proved to be Castleman's disease. We also perform a review of the current literature pertaining to such lesions, focusing on the management of the various clinical and histological variants of the disease.

## 2. Case Presentation

A 40-year-old female was admitted to our Surgical Department due to an asymptomatic left pararenal mass. The lesion was incidentally discovered during routine gynecological ultrasound workup. Past medical history was unremarkable. The patient did not report any abdominal discomfort, flank pain, back pain, weight loss, sweating, fatigue, or fever. On physical examination, she was afebrile with normal vital signs. Superficial lymphadenopathy or organomegaly was absent. Complete blood count, serum biochemistry, and tumor markers (CA 19-9, CA-125, CEA, AFP, and NSE) were all within normal range.

**Figure 1:**
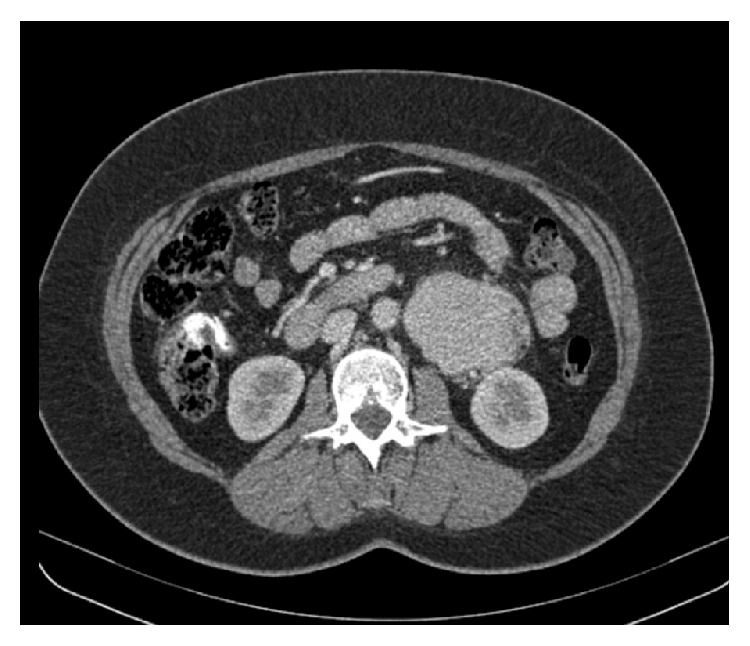
Preoperative abdominal computed tomography (CT) scan depicting a voluminous left pararenal mass.

**Figure 2:**
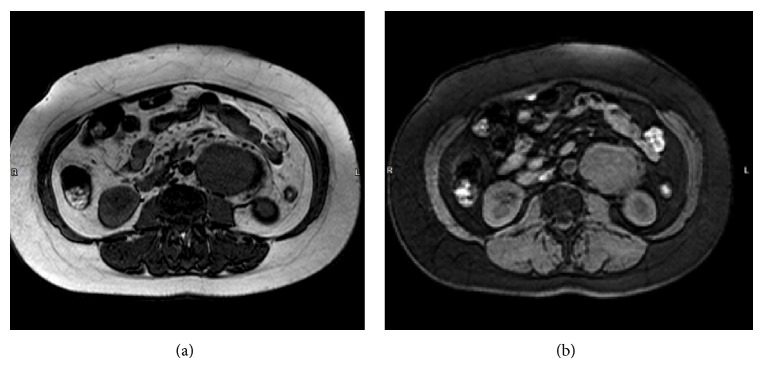
(a), (b) Preoperative magnetic resonance imaging (MRI). A left pararenal mass is shown (T1 and T2 hyperintensity, resp.).

Further investigation with thoracic and abdominal computed tomography revealed a 7.6 × 5.3 × 12 centimeters' heterogeneous lesion adjacent to the left kidney. The mass was hypervascular with regular margins and did not invade other structures. Imaging of the thorax, mediastinum, and the rest of the abdominal cavity was unremarkable (Figure 1). On abdominal magnetic resonance imaging, the mass exhibited both T1 and T2 hyperintensity in a heterogeneous fashion (Figures 2(a) and 2(b)). Based on the imaging modalities, differential diagnosis included malignancy and a lymph nodal mass.

Due to diagnostic uncertainty, we opted for a surgical exploration. The lesion was entirely removed via a left flank approach under general anesthesia. Postoperative course was uneventful and the patient was discharged on the 5th postoperative day.

Histology revealed a lymph node specimen constituted by small-sized follicles with atrophic germinal centers and hyperplastic follicular mantle regions. The germinal centers were almost totally replaced by endothelial and dendritic cells. Mantle cells were arranged in layers surrounding the atrophic germinal centers. The interfollicular area abounded in numerous postcapillary venules, small vessels, with concentric fibrosis or hyalinization of their wall, and multiclonal plasma cells, while the number of the lymphocytes was reduced. No normal lymphoid tissue or lymph node sinus was recognized circumferentially. The immunohistochemical assays highlighted the CD20 expression of the mantle cells surrounding the hyaline vascular germinal center follicles, the dendritic cell hyperplasia, prominent vascularity of the interfollicular area, and reduced expression of Ki67 in the atrophic germinal centers. HHV-8 staining was negative. Histology was conclusive for Castleman's disease, hyaline/vascular variant.

**Figure 3:**
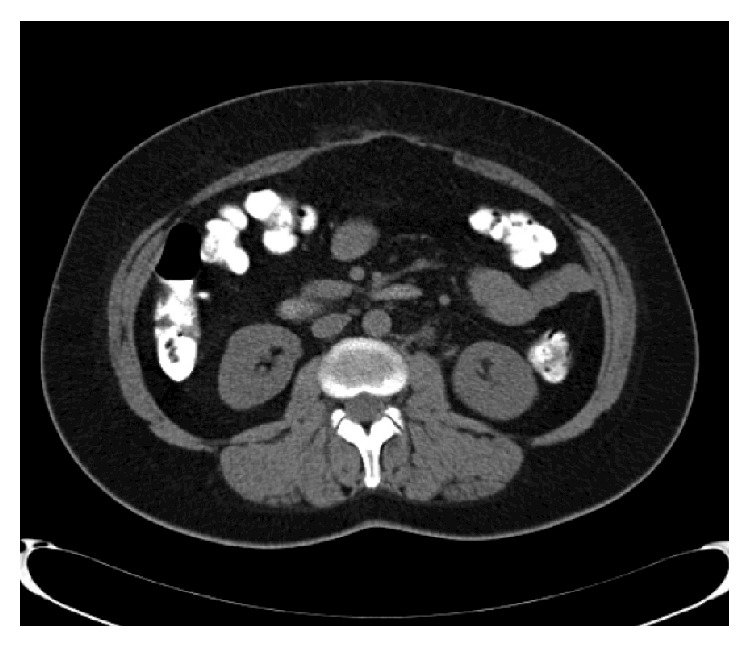
Abdominal computed tomography (CT) scan one year after the operation; the postoperative site is free of recurrent disease.

One year after the operation, the patient remains asymptomatic and in perfect general condition. Yearly abdominal computed tomography was negative for local recurrence or any other intra-abdominal pathology (Figure 3).

## 3. Discussion

The retroperitoneum can host a wide spectrum of pathologies, including a variety of rare benign tumors and malignant neoplasms that can be either primary or metastatic lesions. Retroperitoneal tumors can cause a diagnostic dilemma and present several therapeutic challenges because of their rarity, relative late presentation, and anatomical location, often in close relationship with several vital structures in the retroperitoneal space.

Sarcomas comprise a third of retroperitoneal tumors. Other retroperitoneal neoplasms include primary lymphoproliferative tumors (Hodgkin's and non-Hodgkin lymphoma) and epithelial tumors (renal, adrenal, and pancreas) or might represent metastatic disease from known or unknown primary sites (germ cell tumors, carcinomas, and melanomas).

In order to investigate such tumors, various common tumor markers could be used preoperatively. In our case we used CA 19-9, CA-125, CEA, and AFP.

In men with a retroperitoneal mass, the determination of germ cell tumor markers occasionally enables preoperative distinguishing of primary retroperitoneal germ cell tumors with considerable consequences for management. In this setting, a retroperitoneal tumor should be investigated by specific tumor markers such as alpha-fetoprotein and *β*-human chorionic gonadotropin [1].

In some cases of retroperitoneal tumor, neuron-specific enolase (NSE) can be used as a marker for neuronal tissue and thus offers a diagnostic criterion differing neuroblastoma from, for example, Wilms' tumor [2].

Benign tumors can cause concern and are often an incidental finding during an investigation for unrelated symptoms. The most common benign pathologies encountered in the retroperitoneum include benign neurogenic tumors (schwannomas, neurofibromas), paragangliomas (functional or nonfunctional), fibromatosis, renal angiomyolipomas, and benign retroperitoneal lipomas [1].

Castleman's disease (hereafter depicted as CD) should also be included in the differential diagnosis of any hypervascular and heterogeneous tumor mass in the retroperitoneum [3].

Castleman's disease, also known as angiofollicular lymph node hyperplasia, giant lymph node hyperplasia, or lymphoid hamartoma, is a nonclonal, nonneoplastic lymph node proliferation first described by Castleman and Town in 1954. Castleman and Town reported 13 cases of unicentric hyaline vascular lesions of the chest and described the classic pathologic features of hypervascular lymph nodes with hyalinization of vessels, which form concentric arrangements somewhat reminiscent of Hassall corpuscles in the thymus [4–7]. CD has a peak incidence in females on the third and fourth decade of life [8].

Despite its rarity, CD has recently sparked major research interest because of its association with human herpes virus 8 (HHV-8) and HIV infection, providing a model of initially benign viral disease with cytokine-driven propagation into expanding infected cell pools and potent malignant transformation [6]. In HIV infected subjects, CD commonly presents in the fourth decade of life exhibiting a male predominance. Interestingly, the occurrence of the disease seems not to correlate with CD4 count of HIV viral load [9]. It has been reported that HHV-8 could play a role in the pathogenesis of the plasma cell variant of multicentric CD, especially in HIV-infected patients. This virus is also strongly associated with Kaposi's sarcoma and body cavity-based lymphomas [10]. Chronic inflammation, immunosuppression, autoimmunity, and dysregulation of interleukin-6 production have also been implicated in the pathogenesis of CD [5, 9].

Clinically, there are two distinct types of the disease: the unicentric and multicentric subtype.

It is of pivotal importance to identify unicentric as opposed to multicentric disease at a clinical level in a stepwise approach [11]. Unicentric disease typically affects one lymph node station, although occasionally small, regional, and satellite nodes may be present. Patients are diagnosed incidentally or may have symptoms due to compression of neurovascular sites or other vital structures. 3% of these patients present with systemic complaints and less than 10% (usually patients with plasma cell variant) have disease associated syndromes like myasthenia gravis, erythema nodosum, Horton's arteritis, or Moschcowitz's disease [12].

Histologically, CD consists of three variants: hyaline vascular (HV), plasma cell (PC), or mixed (Mixed). The HV variant is described in 90 per cent of CD cases and is characterized by the proliferation of capillary vessels in germinal centers of lymphatic follicles [13]; germinal centers are concentrically arranged—in an onion peel-like formation—by a mantle zone of small lymphocytes [4]. A prominent vascular proliferation is manifested in the interfollicular regions [9]. The HV variant is typically associated with the unicentric subtype and often exhibits less clinical manifestations and a benign prognosis. On the other hand, the PC variant is characterized by hyperplastic follicles and an interfollicular region containing sheets of plasma cells; it is often associated with the multicentric subtype, presenting with systemic manifestations, such as fever, malaise, weight loss, fatigue, edema, anemia, hypergammaglobulinemia, organomegaly, and POEMS syndrome (polyneuropathy, organomegaly, endocrinopathy, monoclonal gammopathy, and skin changes such as erosive lichen planus) [4, 13, 14]. Between 11 per cent and 30 per cent of patients with POEMS syndrome have multicentric CD, most commonly the HHV-8-positive variant [7]. Contrary to the HV variant, the PC subtype often shows a clinically aggressive and relapsing course [15].

The distribution of localized CD has been reported to be 65 per cent in the mediastinum [16], 16 per cent in the neck, 12 per cent in the abdomen, and 3 per cent in the axilla [4]. Mediastinal CD can mimic thymoma, lymphoma, sarcoma, hemangiopericytoma, neural crest, derived neoplasms such as paraganglioma, neurofibroma, or schwannoma, and chest wall tumors [7]. Other localizations include the mesentery, pelvis, pancreas, vulva, adrenal gland, and retroperitoneum [8].

Ultrasonography usually reveals a hypoechogenic and homogenous mass. Computed tomography shows a solid homogenous hypervascular mass when the tumor diameter is less than 5 centimeters, whereas larger tumors (>5 centimeters), because of necrosis or fibrosis, tend to have more heterogeneous enhancement with a central low-attenuation area. Three patterns of involvement have been described, including a solitary noninvasive mass (most common: 50 per cent of cases), a dominant infiltrative mass with associated lymphadenopathy (40 per cent of cases), and matted lymphadenopathy without a dominant mass (10 per cent of cases) [7]. At magnetic resonance (MR) imaging, the lesions of hyaline vascular CD classically exhibit heterogeneous T1 and T2 hyperintensity compared with skeletal muscle. Prominent flow voids may be seen, which identify the feeding vessels. MR imaging is well suited to depict the extent of disease and the relationship to adjacent structures, although evaluation of calcifications is limited [7]. Imaging findings are often nonspecific, and histologic diagnosis is required in nearly all cases for confirmation. Nondiagnostic findings from repeated needle biopsies should increase the suspicion for Castleman's disease, and an excisional biopsy is often needed to establish the final diagnosis [7].

Curative resection, radiotherapy, steroids, immunotherapy such as interferon-alpha or anti-IL-6 antibodies, and combination chemotherapy such as cyclophosphamide, vincristine, and doxorubicin have all been used to manage the disease. Recently, neoadjuvant therapy with rituximab has been suggested [7]. Complete surgical excision is the treatment of choice for localized, unicentric lesions in any organ domain [11]; cytoreduction of radiotherapy has also been advocated in cases where complete resection is not feasible [9]. In a recent report from Memorial Sloan Kettering Cancer Center, complete resection of unicentric disease was curative for all patients regardless of histologic subtype [17]. Likewise, Keller et al. retrospectively examined 61 patients with unicentric disease who were treated with surgery over a 20-year period. Their study demonstrated that, for patients with unicentric HV-CD, complete resection offered the best chance for cure [18].

Faced with the diagnosis of the multicentric subtype of CD, there is no curative indication for surgery because outcomes are at best similar to those obtained with various forms of immunochemotherapy. At present, the role of the surgeon in cases of multicentric CD should be limited to gaining tissue by an appropriate biopsy and to debulking of dominant foci of multicentric disease in presence of specific organ-related indications [11].

## 4. Conclusion

The retroperitoneum can host a wide spectrum of pathologies, including a variety of rare benign tumors and malignant neoplasms that can be either primary or metastatic lesions. Castleman's disease should always be included in the differential diagnosis of any hypervascular and heterogeneous tumor mass in the retroperitoneum. The localized hyaline vascular form presents as a solitary lesion which can be located mainly in the mediastinum, but also in the abdomen, in retroperitoneum, or in many other locations. Complete surgical excision is curative; recurrences have only been described after incomplete resection.
